# Anterolateral Thigh Flap for Abdominal Wall Reconstruction: A Lifesaving Procedure—A Case Report

**DOI:** 10.1055/s-0044-1801806

**Published:** 2025-02-10

**Authors:** N. Nagaprasad, G. Praveen Harish, Asmat Jahan, Kavya Gaddam, Satwika Dharanikota

**Affiliations:** 1 Department of Plastic and Reconstructive Surgery, Osmania Medical College, Hyderabad, Telangana, India

**Keywords:** abdominal wall defect, pedicled anterolateral thigh flap, reconstruction trauma

## Abstract

It is extremely challenging to deal with a complex full-thickness abdominal wall defect following serious trauma. We aim to share our experience in applying an anterolateral thigh flap in abdominal wall defect reconstruction. This is a retrospective case report of a 40-year-old male patient with a large area of full-thickness defect in the abdominal wall complicated with multiple organ damage identified due to acute trauma. Immediate organ repair surgeries were performed. Meanwhile, the patients underwent complete debridement in the zone of the abdominal wall defect, together with negative pressure wound therapy. Then the appropriate timing was chosen to perform a pedicled anterolateral thigh flap with vastus lateralis muscle for reconstructing a large area of full-thickness defect involving the abdomen. The outcome of the patients was also good. Thus, we conclude that the pedicled anterolateral thigh flap and vastus lateralis muscle flap are feasible to repair full-thickness defect in the abdominal wall and serve as lifesaving options.

## Introduction

Abdominal wall defects (AWDs) usually coexist with multiple abdominal injuries, and the vital signs are unstable in such an acute situation. 1 This may lead to treatment delays in patients with AWDs after trauma. For large-sized full-thickness AWD, autologous tissue transplantation was considered an effective method that could promote local vascularization. An autogenous anterolateral thigh (ALT) flap, a workhorse flap for various wounds, has been used for treating abdominal walls with large skin paddle defects. 2 There exist two types of ALT flaps: pedicled and free. 3 Pedicled ALT flaps are characterized by better blood flow, easy of execution, low incidence of complications, large area of reconstruction, and strong anti-infective ability. In this study, we aim to investigate the efficacy of pedicled ALT flaps for the reconstruction of large AWDs.

## Case Report

**Fig. 1:**
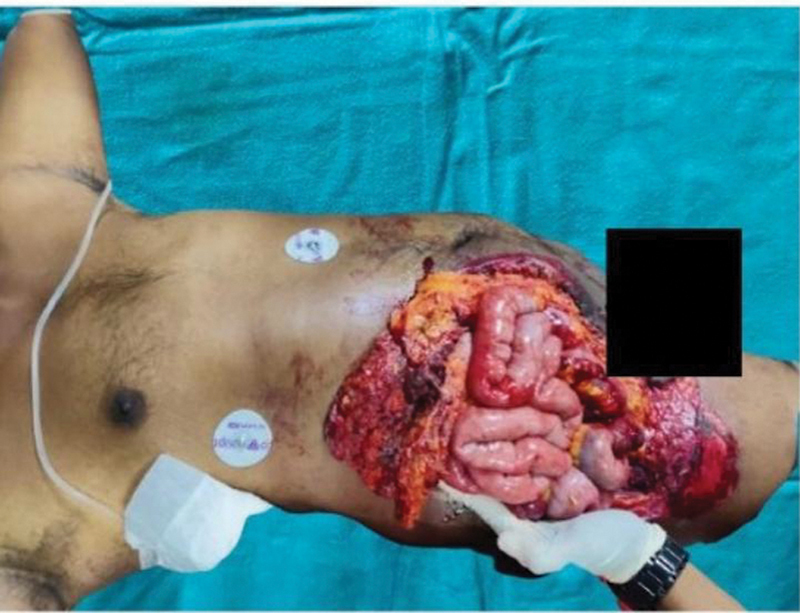
Presentation at the emergency room showing bowel evisceration.

A 40-year-old man sustained injury over the abdomen when a fan blast occurred at a poultry farm and presented at the emergency room with bowel evisceration ( Figs. 1 and 4A ). Immediate emergency bowel exploration and repair were done. Following this, repeated abdominal wall debridement was done and negative pressure wound therapy was applied for three to four sessions.

**Fig. 2:**
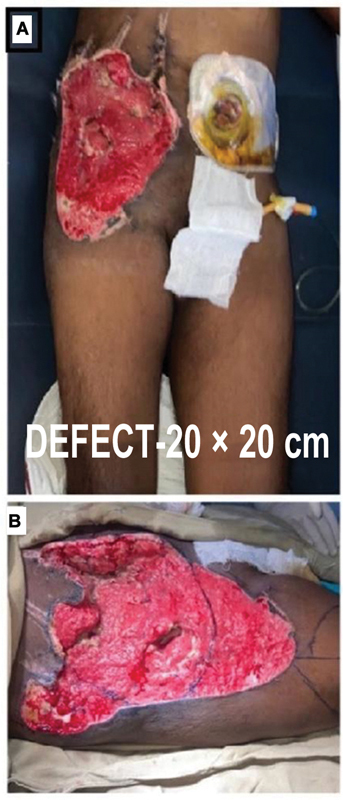
Post-op stoma and post vacuum-assisted closer application showing defect. ( **A** ) The stoma site on postoperative day 25. ( **B** ) Defect over the right iliac fossa, right lumbar, and hypogastrium quadrants of the abdomen.

**Fig. 3:**
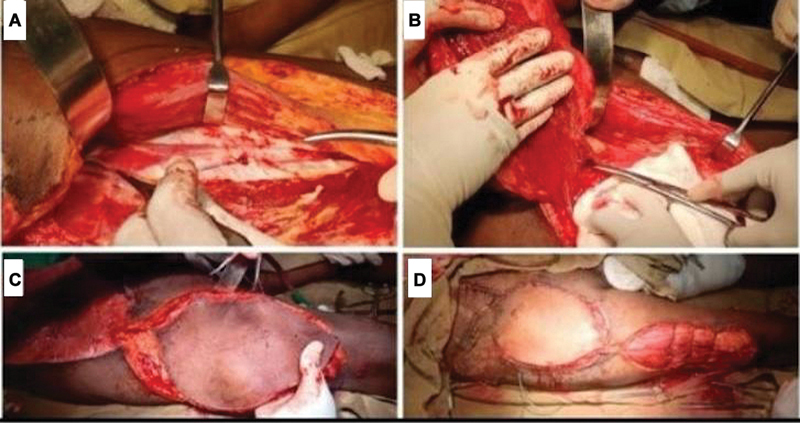
Anterolateral thigh (ALT) flap with vastus lateralis muscle. ( **A** ) ALT flap harvested with the vastus lateralis muscle. ( **B** ) Pedicle of the ALT flap. ( **C** ) ALT flap harvested. ( **D** ) ALT flap covering the abdominal wall defect.

**Fig. 4:**
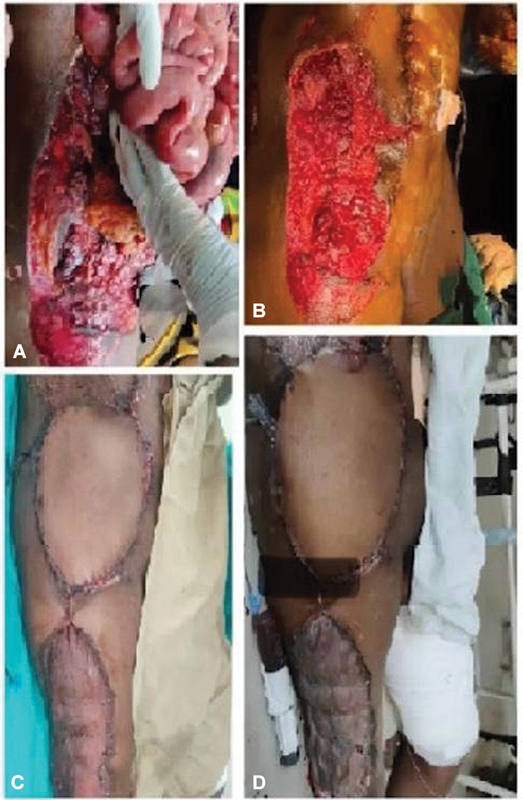
Abdomen wall reconstruction. ( **A** ) Initial bowel evisceration from the abdominal wound. ( **B** ) Defect after vacuum-assisted closer application. ( **C** ) Immediate post-op anterolateral thigh (ALT) flap. ( **D** ) Postoperative day 7 of ALT flap.

Initially, the General Surgery Team explored the abdomen, bowel and mesenteric injuries were addressed, and a loop ileostomy was done. A local advancement flap was tried to reduce the AWD. Bowel contents were covered in a Bogota bag. On postoperative day 4, bowel edema subsided, the bowel was placed back in the abdomen, and the wound was approximated with a thin layer of surrounding tissue. However, there was still muscle loss, necessitating four sessions of negative pressure wound therapy (vacuum-assisted closer [VAC] therapy; Figs. 2B , 4B ) with serial debridement of necrotic tissue over the abdominal wall.

Still, there was a large defect of approximately 20 × 20 cm ( Fig. 2A ) over the right side of the abdomen involving the lumbar region and right iliac fossa quadrants of the abdomen. Twenty-five days after initial surgery, the decision was made to reconstruct the full-thickness abdominal wall with a pedicled ALT flap ( Fig. 3C ). A pedicled myocutaneous ALT flap was designed over the right thigh. The flap was raised along with the vastus lateralis muscle ( Fig. 3A ), and the perforator was dissected to its origin in the descending branch of the lateral circumflex femoral artery and then up to the main circumflex trunk. When enough pedicle ( Fig. 3B ) length of approximately 15 cm was obtained, the flap was rotated 180 degrees over the inguinal ligament to the defect and sutured in place with 3–0 Monocryl and 3–0 Prolene to cover the AWD ( Fig. 3D ). There was raw area of approximately 4 × 7 cm proximal to the defect in the right lumbar region, which was resurfaced with skin graft. The flap donor site was covered with a skin graft.

The postoperative period was uneventful, and the flap was 100% viable ( Fig. 4C ), so the patient was discharged on postoperative day 7 ( Fig. 4D ) of the ALT flap surgery. The patient was instructed to avoid any strenuous work. The patient came for follow-up at 1 week, 15 days, 1 month, 3 months, and 6 months. The flap was healthy with perfect integration and fascial continuity of the abdominal wall and no hernia occurred. The graft uptake was 99% over the flap donor site.

## Discussion

Local options for abdominal wall reconstruction include primary closure, skin grafting, tissue expansion, negative pressure wound therapy, component separation, prosthetic mesh, and pedicled or free flaps. 4

Mathes et al classify AWDs into two categories. Type I defects have an intact skin coverage, whereas type II defects have an unstable or absent skin cover. 1 Defects of the abdominal wall can also be divided into partial thickness or full thickness. Partial-thickness defects can be repaired with primary closure, negative pressure–assisted closure, and skin grafting, while full-thickness defects require musculofascial reinforcement using flaps and mesh.

Due to the abundant soft tissue and laxity of the abdominal wall, most abdominal wounds are amenable to local soft tissue closure. Indications for flap coverage vary by etiology of defect, defect characteristics, and timeline for closure.

Local transposition flaps like deep inferior epigastric artery perforator flap, superficial inferior epigastric artery perforator flap, and keystone flaps were not chosen for abdominal wall reconstruction in our case as there was loop ileostomy on the left side of the lower abdomen.

The tensor fascia lata (TFL) flap is mostly used for smaller defects close to the harvesting point. Sometimes the TFL flap may be used if good perforators for the ALT flap are not found. 5 A regional flap like the pedicled ALT flap was chosen in our case because of its better reliability of blood supply, relative ease of dissection, and pedicle length. It was considered to be the most effective choice for treating AWDs. Moreover, the rich blood supply of the flap makes it more resistant to infection and reduces recovery time. 6 Like in other types of flaps, ALT flaps can be used as either pedicled or free flap. While using a pedicled ALT flap in our case, it was important to tunnel the flap below the rectus femoris muscle proximally to achieve the additional length of the pedicle and tunnel into the abdomen without compressing the pedicle. 7 The long pedicle length also allows a wide arc of rotation, and to an achieve extra length, the branches to the rectus femoris and TFL were divided. In our case, the ALT flap along with the vastus lateralis muscle was chosen to restore abdominal wall integrity, muscle function, abdominal wall strength, and skin coverage for this large defect.

## Conclusion

Abdominal wall reconstruction can be performed using various surgical techniques, among which the ALT and TFL flaps are the most frequently used. 8 The ALT flap is considered to be the most advantageous flap used for abdominal wall reconstruction because it can be used free or pedicled, and it has a large skin paddle, good blood supply, large pedicle length, better arc of rotation, and is relatively easy to dissect. We present a complex case in which a full-thickness abdominal defect was reconstructed using a pedicled ALT flap with the vastus lateralis muscle.
